# Health financing strategies for achieving Universal Health Coverage in low- and middle-income countries: a narrative review

**DOI:** 10.3389/fpubh.2026.1868936

**Published:** 2026-07-17

**Authors:** Sharmake Gaiye Bashir, Yusuf Hared Abdi, Zakaria Ibrahim Issack, Ilyas Abdullahi Khalif, Sumaya Ahmed Ali, Shadia Mohamed Ali, Bashir Mohamed Abdi, Yusuf Abdullahi Hubow, Aniso Mohamed Abdi, Mohamed Sharif Abdi, Naima Ibrahim Ahmed, Yakub Burhan Abdullahi, Gallad Dahir Hassan

**Affiliations:** 1 Faculty of Health Science, Salaam University, Mogadishu, Somalia; 2 Faculty of Health Sciences and Tropical Medicine, Somali National University, Mogadishu, Somalia; 3 Center for Health Research and Innovation, Somali National University, Mogadishu, Somalia; 4 Health Emergency Department, Ministry of Health & Human Service, Mogadishu, Somalia

**Keywords:** financial risk protection, health financing, low- and middle-income countries, out-of-pocket expenditure, Universal Health Coverage

## Abstract

**Background:**

Low- and middle-income countries (LMICs) face persistent health financing challenges, including heavy out-of-pocket payments, fragmented risk pools, and constrained fiscal space that undermine financial protection and equitable access to care.

**Objective:**

This narrative review synthesizes evidence on health financing strategies in LMICs, examining how revenue generation, risk pooling, and strategic purchasing can advance Universal Health Coverage (UHC).

**Methods:**

PubMed/MEDLINE, Scopus, and Web of Science were searched for English-language publications from January 2016 to February 2026, supplemented by policy documents from international health organizations. Of 120 records identified, 58 full texts were assessed and 24 sources were included, comprising systematic reviews, country case studies, and policy analyses across sub-Saharan Africa, South Asia, and Southeast Asia. A formal risk-of-bias appraisal was not applied; instead, sources underwent a structured credibility assessment.

**Results:**

Tax-based financing and subsidized insurance models appear more consistently associated with coverage expansion and financial protection when supported by adequate public funding, mandatory inclusion, and large risk pools. Social health insurance contributes to equity only where enrollment is mandatory and premiums for the poor and informal workers are subsidized. Community-based insurance plays a complementary rather than cornerstone role, while donor funding and innovative mechanisms offer supplementary support but raise concerns about sustainability and scale. Across settings, fragmented, voluntary, and out-of-pocket-dominated arrangements are repeatedly linked to weaker financial protection.

**Conclusion:**

No single financing model is sufficient for UHC. The evidence suggests that integrated, tax-anchored systems with consolidated risk pools, strategic purchasing, and strengthened governance represent the most plausible pathway for LMICs, though financing reform alone cannot guarantee progress, since service readiness, workforce capacity, and governance determine whether financial coverage becomes effective coverage.

## 1 Introduction

Universal Health Coverage (UHC) has emerged as a central pillar of the global health agenda and is enshrined in Sustainable Development Goal (SDG) target 3.8, which calls for access to quality essential health services and financial risk protection for all (1, 2). UHC is commonly defined as ensuring that all people can obtain the promotive, preventive, curative, rehabilitative, and palliative services they need, of adequate quality, without suffering financial hardship (1, 2). Progress towards UHC is particularly urgent in low- and middle-income countries (LMICs), where health needs are high, service coverage is often poor, and reliance on out-of-pocket (OOP) payments exposes households to catastrophic costs and impoverishment (2, 3). Health financing is a fundamental determinant of whether health systems can expand access and secure financial protection (1, 4). It encompasses three core functions: mobilizing sufficient resources, pooling funds to enable risk-sharing, and purchasing services in ways that promote equity and efficiency (2, 4). Evidence from LMICs shows that prepayment and pooling arrangements such as tax-funded systems and social or community-based health insurance are associated with higher service utilization and improved financial risk protection compared with fragmented, OOP-dominated systems (2, 5). Conversely, persistent dependence on OOP spending and donor funding, combined with limited fiscal space, constrains the capacity of LMICs to scale essential services and undermines progress towards UHC (3, 4). Despite growing political commitment LMICs face substantial health financing challenges (3, 6). Many systems are characterized by low and volatile public spending on health, narrow and regressive revenue bases, and a high share of private OOP payments, which remain the predominant source of health financing in Sub-Saharan Africa and other LMICs (3, 4). Fragmentation of financing through multiple, poorly coordinated schemes with distinct pools, benefits, and eligibility criteria reduces cross-subsidization and generates inefficiencies that impede equity and financial protection (1, 7). Efforts to expand social health insurance often struggle to cover large informal sectors, while exemptions and targeted subsidies for the poor are frequently underfunded and weakly implemented, resulting in coverage gaps and continued financial barriers (7, 8). Moreover, competing priorities, governance weaknesses, and rising health care costs complicate the transition to more sustainable and progressive financing arrangements (9, 10). In this context, a critical examination of health financing strategies is essential for guiding reforms that can strengthen health systems and accelerate progress towards UHC (2, 10). Systematic and narrative reviews highlight that no single financing model is universally optimal; instead, context-specific mixes of tax funding, insurance schemes, and demand-side financing mechanisms are needed to enhance coverage, equity, and resilience (2, 8). Understanding how different strategies perform in LMICs in terms of revenue raising, risk pooling, purchasing, and their impacts on utilization, quality, and financial protection is vital for informing policy choices and prioritizing scarce resources (2, 4).

Although recent reviews have catalogued health financing mechanisms in LMICs, most describe these mechanisms in isolation and treat the link between financing and UHC as a direct one. Less attention has been paid to the structural conditions under which a given mechanism succeeds or fails. This review addresses that gap. It asks under what conditions revenue generation, risk pooling, and strategic purchasing translate into financial protection and effective coverage across diverse LMIC settings. Using a conditional, systems-oriented framework in which governance, institutional capacity, and provider behaviour mediate financing outcomes, the review synthesizes evidence across regions and derives policy-relevant lessons for fiscally constrained systems. Its distinctive contribution is to move from cataloguing mechanisms toward explaining the conditions that determine their effectiveness.

## 2 Methods

### 2.1 Study design and reporting approach

This study employed a structured (systematized) narrative review design to synthesize evidence on health financing strategies and their role in advancing Universal Health Coverage (UHC) in low- and middle-income countries (LMICs). Although the review addresses LMICs as a single overarching category, financing options differ across income levels; lower-middle-income settings often have broader tax bases and greater administrative capacity than low-income settings. Where the evidence allows, findings are therefore interpreted with attention to these income-group differences. A narrative approach was selected because the breadth and heterogeneity of health financing evidence — spanning diverse financing mechanisms, health-system architectures, country contexts, and outcome measures — does not lend itself to a single homogeneous search protocol or meta-analytic synthesis. However, to reduce the subjectivity and selection bias commonly associated with traditional narrative reviews, the review was conducted using transparent and reproducible procedures. Reporting was guided by the principles of the PRISMA 2020 statement (adapted for a narrative synthesis) for documenting the search and selection process, and by the SANRA (Scale for the Assessment of Narrative Review Articles) criteria to support methodological quality. The review was organized around three core health financing functions — revenue generation, risk pooling, and strategic purchasing — and their implications for financial protection and equitable access to care.

### 2.2 Literature search strategy

A systematic literature search was conducted in three electronic databases PubMed/MEDLINE, Scopus, and Web of Science (as shown in Table 1). The search was limited to peer-reviewed publications in English published between January 2016 and February 2026, a window selected to capture the most recent decade of financing reforms relevant to current UHC debates while still encompassing foundational contemporary evidence.

**Table 1:** Search strategies applied across the three electronic databases (search date: February 2026).

Database	Field tags	Search string	Limits
PubMed/MEDLINE	MeSH + Title/Abstract [tiab]	(“Healthcare Financing”[Mesh] OR “Insurance, Health”[Mesh] OR “health financing”[tiab] OR “health care financing”[tiab] OR “revenue generation”[tiab] OR “risk pooling”[tiab] OR “social health insurance”[tiab] OR “community-based health insurance”[tiab] OR “strategic purchasing”[tiab] OR “provider payment”[tiab] OR “out-of-pocket payment*”[tiab] OR “catastrophic health expenditure”[tiab]) AND (“Universal Health Insurance”[Mesh] OR “universal health coverage”[tiab] OR “UHC”[tiab] OR “financial protection”[tiab] OR “healthcare access”[tiab]) AND (“Developing Countries”[Mesh] OR “low-income countr*”[tiab] OR “lower-middle-income countr*”[tiab] OR “low- and middle-income countr*”[tiab] OR “LMIC*”[tiab] OR “developing countr*”[tiab])	English; 2016/01/01–2026/02/28; Humans
Scopus	TITLE-ABS-KEY	TITLE-ABS-KEY(“health financing” OR “health care financing” OR “revenue generation” OR “risk pooling” OR “social health insurance” OR “community-based health insurance” OR “strategic purchasing” OR “provider payment” OR “out-of-pocket payment*” OR “catastrophic health expenditure”) AND TITLE-ABS-KEY(“universal health coverage” OR “UHC” OR “financial protection” OR “healthcare access”) AND TITLE-ABS-KEY(“low-income countr*” OR “lower-middle-income countr*” OR “low- and middle-income countr*” OR “LMIC*” OR “developing countr*”)	English; 2016–2026; Article, Review
Web of Science (Core Collection)	Topic (TS)	TS = (“health financing” OR “health care financing” OR “revenue generation” OR “risk pooling” OR “social health insurance” OR “community-based health insurance” OR “strategic purchasing” OR “provider payment” OR “out-of-pocket payment*” OR “catastrophic health expenditure”) AND TS = (“universal health coverage” OR “UHC” OR “financial protection” OR “healthcare access”) AND TS = (“low-income countr*” OR “lower-middle-income countr*” OR “low- and middle-income countr*” OR “LMIC*” OR “developing countr*”)	English; 2016–2026; Article, Review

### 2.3 Eligibility criteria

Studies were selected through an iterative, purposive process guided by their relevance to the review’s themes and objectives. The following criteria informed source selection throughout the review process.

#### 2.3.1 Inclusion criteria

Sources were considered for inclusion if they: were published in peer-reviewed academic journals or were policy documents and technical reports produced by internationally recognized health organizations; examined health financing mechanisms or strategies relevant to Universal Health Coverage; focused on low- and middle-income countries (LMICs); reported findings related to financial protection, healthcare access, service utilization, or health system financing reforms; and were available in English.

#### 2.3.2 Exclusion criteria

Sources were excluded if they: were editorials, commentaries, or opinion pieces without empirical or policy evidence; did not address health financing or UHC-related outcomes; focused exclusively on high-income country settings; or were non-peer-reviewed sources of unclear provenance, conference abstracts without full publications, or sources without sufficient methodological transparency.

### 2.4 Study selection process

**Figure 1:**
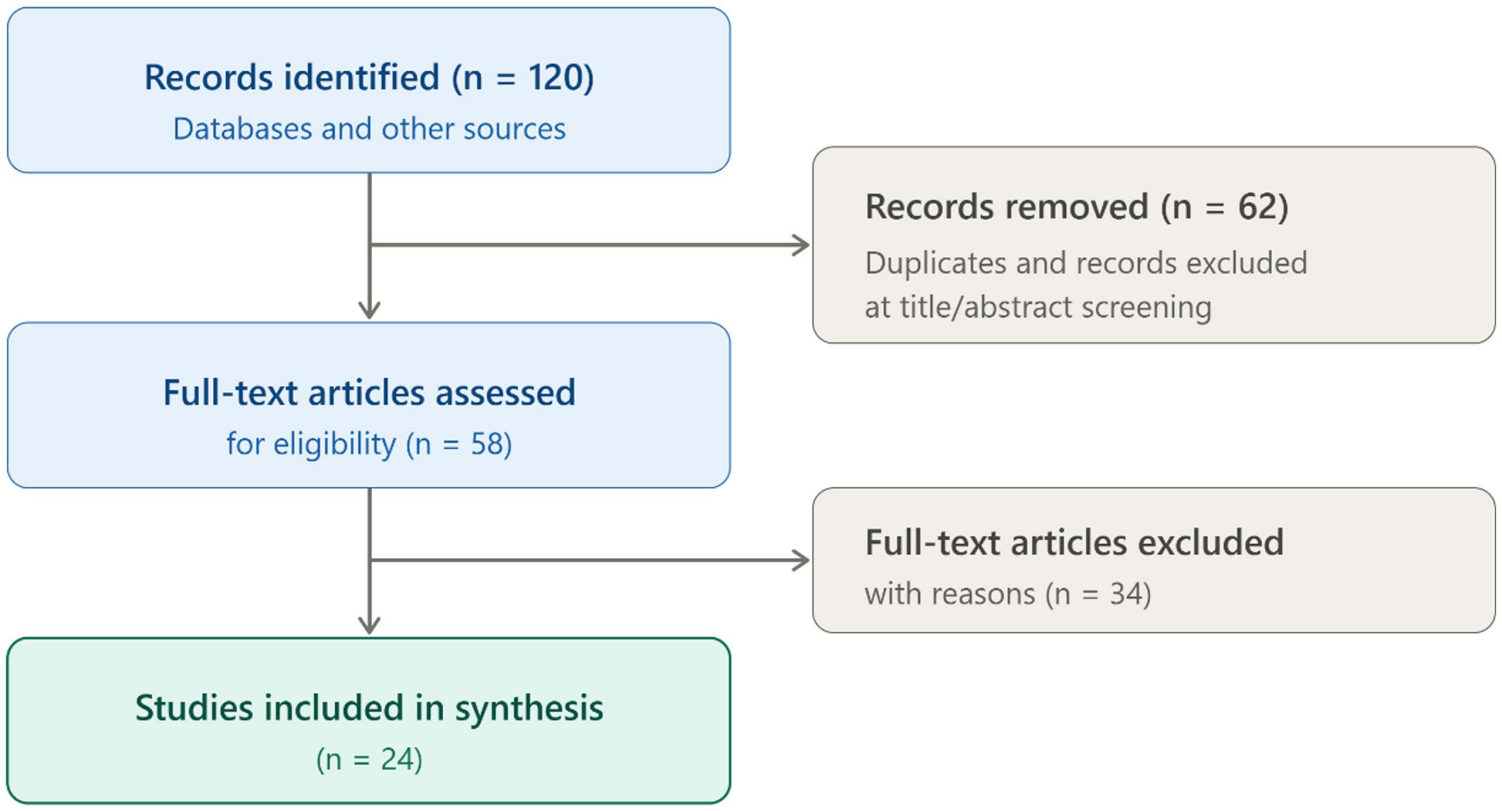
PRISMA flow diagram illustrating the study selection process for the narrative review.

Source selection followed an iterative and purposive process suited to narrative review methodology. The database search identified 120 records. Following the removal of duplicates and screening of titles and abstracts, 58 full-text articles were assessed for eligibility, of which 34 were excluded for not meeting the inclusion criteria. Sources that appeared pertinent at the title and abstract stage were read in full and assessed against the review’s three core functions — revenue generation, risk pooling, and strategic purchasing — as well as coverage of financial protection and equity outcomes. A total of 24 sources were ultimately included in the synthesis, comprising systematic reviews, narrative reviews, country case studies, and policy analyses. The study selection process is summarized in Figure 1. A distinction is drawn between sources included in the thematic synthesis and those cited for background or contextual purposes. The 24 sources reported here constitute the evidence base that was systematically charted and synthesised against the review’s three core financing functions. Additional references cited in the Introduction and Discussion provide conceptual or contextual support and were not part of the charted synthesis sample.

### 2.5 Quality and credibility assessment

Because the review integrated heterogeneous evidence types (empirical studies, reviews, and policy reports), a single formal risk-of-bias instrument (e.g., GRADE or meta-analytic appraisal) was not appropriate for the full sample. Instead, each source underwent a structured credibility assessment, in which the following were considered: (a) peer-review status or institutional authority of the publishing body; (b) transparency and appropriateness of the underlying methods; (c) relevance and directness of the evidence to LMIC settings; and (d) consistency of findings with the broader evidence base. Greater interpretive weight was given to peer-reviewed empirical studies and evidence from internationally recognized organizations, while lower-credibility or weakly transparent sources were treated with appropriate caution during synthesis. This approach preserves the interpretive flexibility of the narrative method while guarding against the implicit assumption that all sources are equally credible.

### 2.6 Data charting

Key information was charted from each included source to support thematic organization of the evidence. For each source, the following details were noted: study setting and country context; type of health financing mechanism examined; financing model or intervention; key outcomes related to UHC (e.g., financial protection, service utilization, healthcare access); and policy implications and reported challenges. This information was organized thematically rather than in a formal extraction table, in keeping with the iterative and interpretive nature of the narrative review approach.

### 2.7 Data synthesis

**Figure 2:**
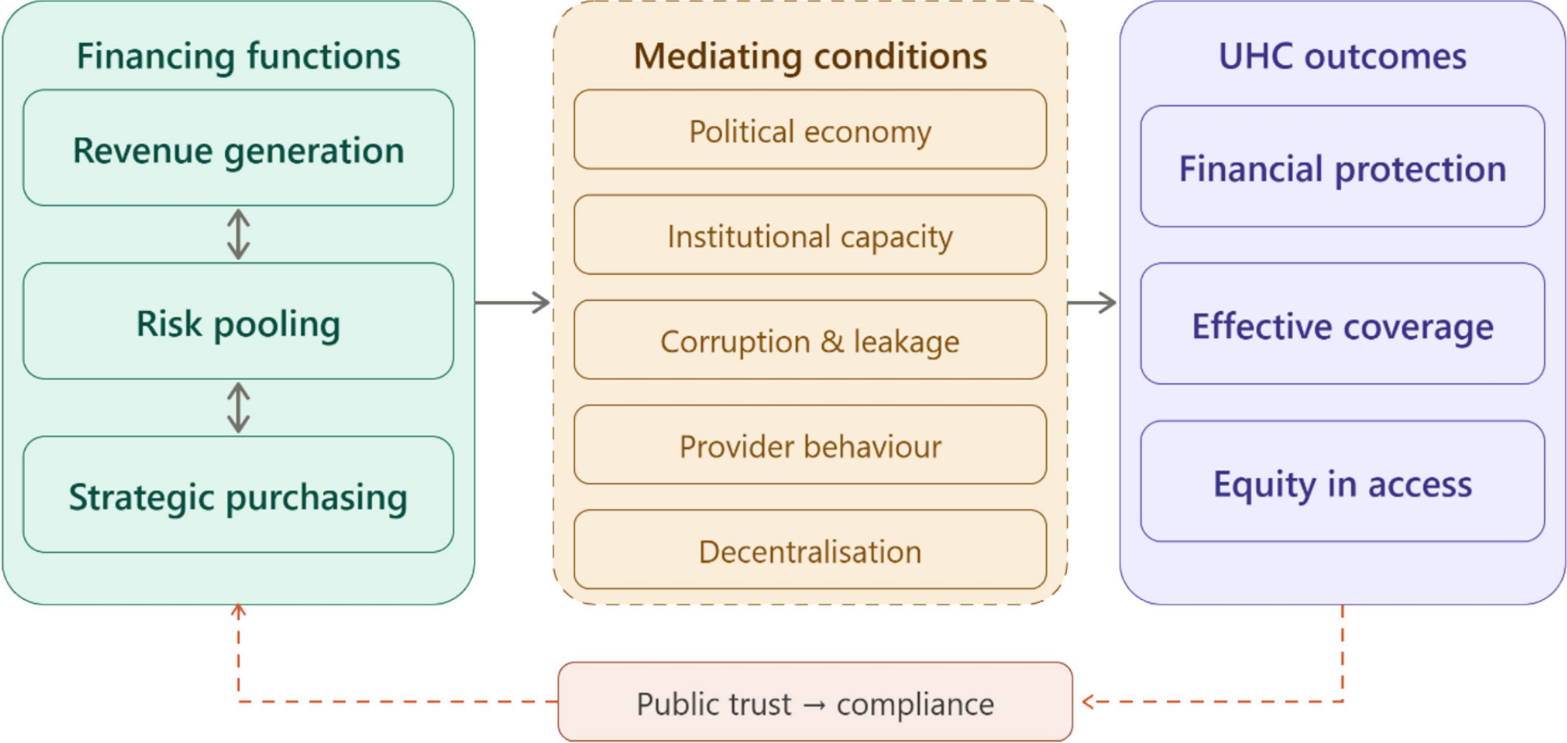
Dynamic conceptual framework of health financing and UHC in low- and middle-income countries.

A thematic narrative synthesis approach was used to analyze and integrate findings from the included sources. Analysis was guided by the conceptual framework presented in Section 4 (Figure 2), which maps the three core financing functions — revenue generation, risk pooling, and strategic purchasing — onto UHC outcomes while accounting for the mediating conditions that shape them, thereby providing a structured lens through which evidence was interpreted and organized. Evidence was grouped according to major health financing functions and policy themes, with the synthesis focusing on tax-based financing, social health insurance, community-based health insurance, donor funding, and innovative financing strategies. Themes were further analyzed in relation to their impact on UHC outcomes, including financial protection, health service accessibility, and equity in healthcare financing. This approach allowed the identification of recurring patterns, policy implications, and gaps in the current evidence base.

## 3 Conceptual framework

Figure 1 illustrates a linear and reinforcing pathway where diverse health financing mechanisms—ranging from domestic tax-based systems and social health insurance to external donor assistance—serve as the primary inputs for the health system. These mechanisms feed into the three core health financing functions: revenue collection aggregates financial resources, risk pooling spreads financial risk across the population to prevent catastrophic out-of-pocket expenditures, and strategic purchasing ensures that resources are allocated efficiently to providers. The effective execution of these functions drives critical health system outcomes, specifically by expanding equitable access to services, ensuring financial risk protection, and enhancing the quality of care. Collectively, these intermediate outcomes culminate in the achievement of Universal Health Coverage (UHC), where all individuals receive the health services they need without suffering financial hardship.

A systems-oriented conceptual framework for health financing and UHC in LMICs (Figure 2). The three core financing functions operate as an interacting subsystem rather than a linear sequence. Their influence on UHC outcomes (financial protection, effective coverage, and equity) is mediated and moderated by contextual conditions — political economy, institutional capacity, corruption and leakage, provider behaviour, and decentralisation. A feedback loop runs from outcomes back to financing: improvements in financial protection strengthen public trust and perceived legitimacy, which raise contribution compliance and, in turn, revenue generation and pooling capacity. This conditional, feedback-driven structure replaces the unidirectional financing-to-coverage model and underpins the typology of financing transition pathways developed in Section 3.

## 4 Conceptual foundations of health financing

Health financing is a core function of the health system concerned with how financial resources are mobilized, pooled, and used to purchase health services so that populations can obtain needed care without suffering financial hardship (11, 12). It underpins efforts to achieve Universal Health Coverage (UHC), defined as equitable access to quality services with financial protection, and is therefore central to health system performance in low-income countries (12, 13). Conceptually, most contemporary frameworks, building on Kutzin and colleagues, converge on three interlinked functions: revenue generation, pooling of funds (and risks), and strategic purchasing of health services (11, 12).

### 4.1 Revenue generation

Revenue generation (or revenue raising) refers to the collection of funds from various sources general and earmarked taxes, social contributions, insurance premiums, external assistance, and out-of-pocket (OOP) payments to finance the health system (12, 14). For progress toward UHC, the adequacy, stability, and compulsory nature of these revenues are more important than the sheer number of financing instruments (12, 13). Evidence from low- and middle-income settings shows that countries advancing fastest toward UHC tend to rely predominantly on compulsory public funding (taxes and mandatory contributions), with OOP payments kept to a low share of total health expenditure (13, 15). This works by separating payment from the point of use. When revenue is raised in advance through compulsory taxes or contributions, care is paid from pooled prepaid funds rather than from a patient’s own pocket at the moment of illness, removing the price barrier that deters care-seeking and shields households from catastrophic costs. Financial protection therefore depends less on total spending than on the share that is prepaid and compulsory rather than paid directly by patients. In low-income countries, however, fiscal space is constrained and OOP spending remains high, often exceeding levels consistent with financial protection and contributing to catastrophic and impoverishing health expenditure (3, 16). Thus, the core conceptual challenge in revenue generation is to increase prepaid, progressive public funding while reducing reliance on OOP payments that act as a barrier to access and undermine equity (15, 17).

### 4.2 Pooling of funds and risk

Pooling is the accumulation and management of prepaid health funds so that the financial risk of illness is shared across population groups, decoupling the use of services from individuals’ ability to pay at the point of care (11, 12). Conceptually, pooling transforms fragmented individual expenditures into collective resources that can be allocated according to need rather than income or health status. The redistributive capacity of a health financing system depends critically on how funds are pooled: a given level of funding organized into fewer, larger pools has greater potential for cross-subsidization from rich to poor, healthy to sick, and economically active to inactive than the same resources divided into many small, segmented schemes (11, 12). In many low-income and lower-middle-income settings, multiple, uncoordinated pools exist for different employment, geographic, or social groups, leading to duplication, inequities in benefit entitlements, and administrative inefficiency (15, 16). Fragmented pooling arrangements are associated conceptually, and increasingly empirically, with weaker financial protection and persistent inequities in service use, whereas efforts to merge or harmonize pools are viewed as key reforms for advancing UHC (15, 18).

### 4.3 Strategic purchasing of health services

Purchasing concerns how pooled funds are translated into services what is bought, from whom, and how providers are paid (12, 14). Strategic purchasing contrasts with passive payment of historical budgets or fees, and involves deliberate decisions to allocate resources in ways that promote efficiency, quality, and equity in service delivery (19, 20). This includes defining and revising benefit packages or essential service packages, selecting and accrediting providers, setting provider payment methods (for example, capitation, diagnosis-related groups, performance-based financing, or mixed models), and linking payment to performance and population needs (12, 21). Each payment method transmits a different incentive. Capitation pays a fixed amount per enrolled person, encouraging cost control and prevention but risking under-provision unless quality is monitored. Output-based payments reward each service delivered, expanding activity but potentially driving over-provision and cost escalation. Global budgets fix provider income in advance, improving cost control but blunting incentives for volume if set too rigidly. Because no single method aligns behaviour perfectly, many systems use blended models combining a stable base payment with performance-linked components. Nominal coverage becomes effective coverage only when payment design actively steers providers toward needed services rather than passively reimbursing whatever is delivered. Evidence from low- and middle-income countries indicates that when purchasing is aligned with UHC objectives through, for instance, results-based financing, capitation with quality safeguards, or targeted subsidies for vulnerable groups it can improve service availability and utilization among the poor, reduce delays in procurement, and enhance responsiveness of providers (20, 21). Conversely, poorly designed provider payment mechanisms and fragmented purchasing arrangements may encourage cost escalation, compromise quality, or reinforce inequities (12, 19).

### 4.4 Health financing functions, health system performance, and UHC

The three financing functions work together to shape health system performance across key UHC dimensions: population coverage, service coverage, and financial protection (12, 13). Adequate, predominantly compulsory revenue generation creates the fiscal basis for expanding coverage; effective pooling determines the extent to which these resources can be redistributed to protect high-need and low-income groups; and strategic purchasing translates financial resources into accessible, high-quality services that address priority health needs (12, 13). High-performing health financing for UHC is characterized by sufficient and sustainable prepaid funding, large and coherent risk pools, and purchasing arrangements that align provider incentives with efficiency, equity, and quality (12, 13). Empirical syntheses from Africa and Asia show that systems relying heavily on general revenues and mandatory insurance contributions, with explicit political commitment to universalist approaches and subsidization of premiums for the poor and informal workers, achieve broader population coverage and downward trends in OOP spending and catastrophic expenditure (15, 16). In contrast, systems with persistent fragmentation in pooling and purchasing, and continued dependence on OOP payments, exhibit gaps in financial risk protection and inequitable utilization of services, especially at higher levels of care (3, 16). For low income countries, these conceptual foundations imply that health financing reforms must be viewed not as isolated technical fixes but as mutually reinforcing changes in how revenues are raised, pooled, and used to purchase services (12, 18). Progress toward UHC therefore depends on expanding fiscal space through progressive and compulsory funding, consolidating pools to enhance solidarity, and institutionalizing strategic purchasing so that every unit of spending advances equity, efficiency, and quality of care (12, 13). This account, however, describes the financing functions largely as a sequence, and in doing so captures only part of the picture. It says less about how the functions interact, why financing arrangements that appear similar produce divergent outcomes across settings, and how UHC outcomes in turn feed back into the financing system. The following section addresses these questions by setting out a more dynamic, conditional formulation of the financing–UHC relationship.

### 4.5 A conditional, systems-oriented framework

The three financing functions are often presented as a linear sequence in which revenue feeds pooling, pooling feeds purchasing, and purchasing produces UHC. This depiction obscures two features that are central to explaining why similar reforms succeed in some low- and middle-income countries but stall in others. First, the functions operate as an interacting subsystem rather than a one-way chain: the dominant revenue source conditions how funds can be pooled, pooling structure determines the scope available to purchasers, and purchasing arrangements shape the incentives that influence revenue itself. Second, the link between financing and UHC outcomes is not direct but conditional, mediated by five contextual factors — political economy, institutional and administrative capacity, corruption and leakage, provider behaviour, and decentralisation — which together explain why arrangements that appear similar on paper diverge so widely in practice.

This reframing also shifts the relevant outcome from nominal coverage to effective coverage, since expanding entitlement without improving the quality and appropriateness of care yields limited health gains. Finally, the framework incorporates a feedback dynamic that the linear model omits: where reforms deliver visible financial protection, they strengthen public trust and legitimacy, which raise contribution compliance and, in turn, revenue and pooling capacity — while the reverse erodes the financing base. As shown in Figure 2, this conditional, feedback-driven structure replaces the unidirectional financing-to-coverage model and underpins the typology of financing transition pathways developed in the following section.

## 5 Health financing strategies in low-income countries

Low-income countries (LICs) rely on a mix of public, contributory, community-based and external sources to finance health systems, each with distinct implications for equity, efficiency and progress toward Universal Health Coverage (UHC) (2, 3). Evidence shows that no single mechanism is sufficient; instead, adaptive combinations and reforms in revenue raising, pooling and purchasing are required to expand coverage and financial protection in fiscally constrained settings (2, 22). These are strategies also shown in Table 2.

**Table 2:** Major health financing strategies for achieving Universal Health Coverage (UHC) in low-income countries.

Mechanism	Key characteristics	Main strengths	Core limitations	Citations
Tax-based financing	Funded through general government revenue (income tax, VAT); services often provided free at the point of care.	Progressive prepayment; broad pooling; supports subsidies and universalist entitlements	Limited fiscal space, competing priorities, underfunding and high OOPs	(3, 8, 22)
Social/national health insurance	Compulsory contributions from employees/employers (payroll taxes); funds are pooled in a semi-autonomous agency.	Structured pooling; potential equity gains; increased service use	Large informal sector; low contributory capacity; fragmentation; limited coverage of poor	(8, 24, 36)
CBHI/MHI	Voluntary, member-run schemes focused on rural or informal groups; small-scale risk pooling.	Local responsiveness; reduced OOPs and catastrophic spending for enrollees	Small, voluntary pools; weak cross-subsidy; limited benefits; sustainability concerns	(24, 36, 42)
Donor funding	External grants or concessional aid from global agencies (e.g., Global Fund, USAID) for specific health programs.	Rapid program scale-up; augments constrained budgets	Volatility, disease-specific focus, weak alignment with UHC; sustainability risks	(3, 22)
Innovative/earmarked taxes	Targeted mechanisms such as “sin taxes” (tobacco/alcohol), health bonds, or debt-for-health swaps.	Additional domestic revenue; can improve health behaviors	Low revenue yield; political resistance; weak earmarking and implementation capacity	(10, 43)

### 5.1 Tax-based financing

General tax revenues and budgetary allocations remain the backbone of health financing in many LICs, particularly where large informal sectors limit contributory schemes (12, 23). Tax-based financing allows compulsory, potentially progressive prepayment, broad risk pooling and delinking of entitlement from contribution, making it well suited for covering the poor and informal workers (2, 8). Countries such as Thailand and several African UHI schemes that rely heavily on government revenue transfers demonstrate improved coverage and financial protection when tax funding is substantial and stable (2, 24). However, weak tax bases, macroeconomic volatility, competing sectoral priorities and low health spending (often near 2% of GDP) constrain fiscal space, leading to persistent underfunding, benefit rationing and high out-of-pocket payments (OOPs) (10, 12). Policy priorities include raising public spending toward at least 5% of GDP, improving revenue progressivity, and enhancing budget execution and strategic purchasing to convert public funds into effective coverage (10, 12).

### 5.2 Social health insurance

Social and national health insurance (SHI/NHI) have been widely promoted as routes to UHC, particularly in Africa and Asia (3, 25). When designed with mandatory enrollment, integrated pooling and substantial budget transfers for non-contributing groups, SHI can expand coverage and financial risk protection, including for informal workers (8, 26). Insurance-based financing is generally more equitable than direct payments, especially when contribution rates are income-related and benefit packages are comprehensive (25, 27). Nonetheless, LICs face structural constraints: large informal sectors, low contributory capacity and administrative weakness limit payroll-based revenue, resulting in fragmented, often voluntary schemes with low coverage of the poor and persistent OOPs (3, 28). Evidence from African UHI and SHI reforms indicates that contribution-based models alone are unlikely to achieve UHC; robust tax funding and explicit subsidies for informal and vulnerable groups are essential (24, 25).

### 5.3 Community-based and micro health insurance

Community-based and micro health insurance (CBHI/MHI) schemes have proliferated in LICs as locally organized responses to OOP-driven impoverishment (3, 29). They can reduce catastrophic spending and protect assets for enrolled low-income households, and may improve utilization of basic services (30, 31). However, voluntary enrollment, small and fragmented risk pools, adverse selection and limited benefit packages substantially constrain their capacity to provide comprehensive, sustainable financial protection or meaningful cross-subsidization (24, 29). Comparative reviews in Africa show that government-run or national schemes achieve better pooling and management than CBHI, reinforcing the need to integrate or transform CBHI into larger compulsory pools supported by public subsidies (3, 24). Policy implications include using CBHI as transitional or complementary mechanisms linked to national systems through harmonized benefits, reinsurance and targeted subsidies rather than as stand-alone paths to UHC (2, 30).

### 5.4 Donor funding

External assistance remains a major revenue source in many LICs, particularly in sub-Saharan Africa, where donor funding commonly coexists with high OOPs and underdeveloped prepayment mechanisms (2). Donor resources have enabled rapid scale-up of priority programs (e.g., HIV, maternal and child health) and can temporarily ease domestic fiscal constraints (2, 6). Yet aid is often disease-specific, off-budget and unpredictable, undermining country ownership, budgetary planning, and alignment with UHC priorities (2, 3). Heavy reliance on donors also exposes LICs to volatility and jeopardizes sustainability as official development assistance plateaus or declines (2, 3). Strategic policy directions involve progressively substituting external with domestic resources, integrating donor flows into national planning and pooling arrangements, and reorienting aid to strengthen system-wide functions and fiscal space rather than parallel programs (2, 6).

### 5.5 Innovative and earmarked financing mechanisms

LICs have increasingly experimented with “innovative” domestic mechanisms such as sin taxes (on tobacco, alcohol, and sugar-sweetened beverages), mobile phone levies, airline ticket taxes and extractive industry levies to expand fiscal space for health (12, 26). In the WHO African Region, excise taxes on tobacco and alcohol are now nearly ubiquitous, with growing adoption of sugar-sweetened beverage taxes, HIV/AIDS levies, and mobile phone taxes (8, 12). These instruments can generate additional revenues while reinforcing public health objectives by discouraging harmful consumption (8, 26). Evidence suggests, however, that the absolute revenue potential is modest (often below 0.5% of GDP), earmarking for health is rare or partial, and implementation faces strong industry resistance and institutional capacity constraints (12, 26). Political economy, legal design and intersectoral coordination are critical to ensure that such instruments are progressive, efficient and aligned with UHC goals (22, 26).

### 5.6 Synthesis and policy implications

Across mechanisms, key messages are consistent (2, 3). First, reducing reliance on OOPs widely recognized as the most inequitable financing form is essential for financial protection (2, 31). Second, UHC in LICs is most plausibly achieved through a predominantly tax-funded system complemented by contributory insurance for those with ability to pay, underpinned by mandatory coverage, integrated risk pools and explicit subsidies for the poor and informal workers (8, 25). Third, CBHI, donor funding and innovative mechanisms should be leveraged strategically: CBHI as transitional and integrable; aid as catalytic and system-strengthening; and sin and earmarked taxes as incremental, health-promoting revenue sources rather than core pillars (2, 3). Finally, all financing reforms must be coupled with improvements in governance, purchasing and service delivery to translate additional resources into equitable access and better health outcomes (2, 10).

## 6 Challenges to effective health financing

Progress toward Universal Health Coverage in low-income countries is constrained by interlocking structural and systemic barriers that undermine resource mobilization, risk pooling, and financial protection. High out-of-pocket (OOP) expenditures, limited fiscal space, fragmented and shallow risk pools, governance weaknesses, and the predominance of informal employment interact to reproduce inequity and underfunding despite policy commitments to UHC (32). High OOP payments remain a defining feature of health financing in many low-income settings and are a central obstacle to financial protection. In a large group of mostly low-income countries, direct OOP spending has exceeded 50% of total health expenditure, exposing households to catastrophic costs and impoverishment when facing illness (32). A systematic review of catastrophic health expenditure (CHE) shows that heavy reliance on OOP is consistently associated with increased CHE and impoverishment, with social and economic context shaping vulnerability but with financial barriers as the proximate driver of foregone care and poverty transitions (33). Even under insurance regimes, insured clients in several sub-Saharan African countries still make formal and informal payments for services that should be covered, reflecting implementation gaps, resource constraints, and perverse provider incentives; such “double payment” undermines trust and the protective function of prepayment schemes (34). This phenomenon illustrates why nominal coverage does not guarantee financial protection. When insured patients still pay for covered drugs that are out of stock, for diagnostics not actually financed, or in informal charges to providers, the entitlement exists on paper but not in practice. Double payment therefore represents a failure of effective coverage rather than of enrolment, and it persists wherever facility underfunding, supply shortages, and weak claims oversight coexist with formal insurance. Its effect is corrosive, because households who have already prepaid experience the additional charge as a breach of trust, which in turn depresses willingness to enrol and contribute. Persistent OOP dominance therefore signals both inadequate prepayment and weak enforcement of entitlements.

### 6.1 Structural barriers

Limited fiscal space for health further constrains the shift away from OOP financing. Reviews of financing in Africa and Asia highlight chronically low public and total health expenditure as key barriers to UHC, with many countries allocating well below recommended thresholds to the sector (4, 10, 15). In Nepal, for example, total health expenditure remains around 2% of GDP, with more than half of spending coming from OOP and only modest progress on financial protection and service coverage (10). Across ASEAN countries, financial constraints linked to low overall government spending on health are identified as major impediments to expanding coverage and benefits, even where policy frameworks are in place (10). Constrained tax bases, competing fiscal priorities, and limited capacity to mobilize progressive revenues restrict governments’ ability to subsidize coverage for the poor and informal workers, leading to underfunded schemes and narrow benefit packages (2, 10, 15). The large and heterogeneous informal sector in many low-income economies is both a structural and operational barrier to effective health financing. Extending contributory social health insurance to informal workers has proven particularly difficult: a systematic review of Southeast Asia finds that schemes relying on voluntary contributions tend to achieve low and unstable coverage of informal workers, with frequent lapses and regressive patterns of enrollment, whereas universalist, tax-subsidized approaches attain higher coverage and better equity (8). Retrospective policy analysis of Kenya’s National Health Insurance Fund similarly identifies the predominantly informal labor market where 83% of workers lack stable payroll contributions as the major constraint to expanding the SHI pool, resulting in only 17% population coverage and particularly low uptake among informal workers (35). Multiple country experiences confirm that voluntary, contribution-based models in highly informal contexts generate small pools, adverse selection, and persistent exclusion of the poorest, unless combined with substantial tax financing and automatic, mandatory enrollment (2, 8, 36). The informality challenge also intersects with weak identification systems, enforcement limits, and fluctuating incomes that make regular contribution collection administratively costly and politically sensitive.

### 6.2 Implementation barriers

Weak and fragmented risk pooling mechanisms compound these fiscal structural. Multiple, uncoordinated insurance schemes, parallel donor-funded programs, and segmentation between public and private coverage create small, risk-segregated pools that are inequitable and inefficient (2, 15, 37). A systematic review protocol on financing fragmentation underscores that such fragmentation is pervasive in LMICs and is believed to hinder equity, efficiency, and progress toward UHC, even though robust impact estimates remain limited (37). In Nepal, numerous social health protection schemes coexist with low coverage, fragmented pooling, and low efficiency, while the national health insurance faces low enrollment, high dropout, and financial unsustainability as provider payments outstrip contributions (10). In Africa, universal health insurance initiatives are frequently characterized by voluntary enrollment and high fragmentation, features that are considered fundamentally incompatible with universal, equitable coverage (36). Fragmentation thus inhibits cross-subsidization between rich and poor, healthy and sick, and obstructs strategic purchasing.

Governance weaknesses and accountability deficits further undermine effective financing. A narrative synthesis of OOP payments under insurance in sub-Saharan Africa links ongoing informal fees and policy–practice gaps to resource constraints, conflicting policy objectives, and entrenched “practical norms” among frontline staff, as well as to broader corruption dynamics (34). More generally, financing reforms and insurance schemes in LMICs are often hampered by poor stakeholder engagement, politicized decision-making, weak regulation of providers, and limited transparency in purchasing and payment arrangements, leading to inefficiencies, elite capture, and inequitable benefit distribution (2, 38, 39). Scoping reviews of health system governance show that although governance interventions can improve quality and access, the evidence base is thin and many initiatives suffer from inadequate capacity, unclear mandates, and insufficient monitoring (38, 39). Such governance challenges erode confidence in health financing institutions, weaken enforcement of entitlements, and deter enrollment in contributory schemes.

These challenges are mutually reinforcing (as shown in Table 3): low fiscal space leads to underfunded schemes and persistent OOP; fragmentation and weak governance prevent effective pooling and purchasing; and a large informal sector skews financing toward voluntary and regressive mechanisms. Addressing them requires comprehensive reforms that strengthen domestic revenue mobilization, consolidate and tax-subsidize risk pools, tackle corruption and implementation gaps, and adopt inclusive strategies tailored to informal workers, rather than piecemeal adjustments to existing, structurally constrained arrangements (2, 8, 10).

**Table 3:** Key barriers to health financing for Universal Health Coverage.

Barrier	Description	Impact on health system	Evidence from literature	References
Out-of-pocket payments (OOP payments)	Direct fee-for-service charges paid by households at the point of care, often representing the primary source of health funding in low-income settings.	OOP payments lead to catastrophic health expenditures, impoverishment, and significant barriers to service utilization, particularly for vulnerable populations.	Empirical studies in the DRC and Ethiopia confirm that unpredictable OOP payments for primary care result in high household burdens and limited financial risk protection.	(16, 44, 45)
Limited fiscal capacity	Low levels of government revenue and restricted fiscal space for health, often exacerbated by macroeconomic instability or debt.	Constrains the ability to scale up public health investments, forcing a reliance on external donor funding and keeping public service quality low.	Research indicates that countries with low domestic revenue struggle to reprioritize budgets toward health, especially under post-pandemic economic pressures.	(46)
Weak risk pooling mechanisms	Small, voluntary, or disconnected insurance arrangements that fail to effectively distribute financial risk across a broad population.	Reduces cross-subsidization from the healthy to the sick, leading to inequitable benefit packages and inadequate financial protection for the poor.	Cross-country analyses show that incomplete risk-sharing in voluntary schemes prevents the achievement of national-level financial protection goals.	(16, 45)
Fragmented financing systems	The existence of multiple, parallel, and uncoordinated financing pools (e.g., separate donor, civil service, and community schemes).	Creates administrative inefficiencies, duplicates costs, and entrenches health inequities by providing different entitlements based on the pool.	Comparative policy reviews suggest that fragmentation hinders the consolidation of resources needed for a coherent national UHC strategy.	(2, 45)
Governance and accountability challenges	Weak stewardship, lack of transparency, corruption, and insufficient regulatory oversight in the health sector.	Undermines the efficient use of limited resources, weakens purchasing power, and erodes public trust in health financing reforms.	Institutional analyses correlate high levels of corruption and weak political leadership with lower public health spending and poor reform implementation.	(46, 47)
Large informal sector	A predominant workforce engaged in non-payroll activities, making it difficult to enforce mandatory insurance contributions.	Limits the reach of contributory health insurance models, leaving a large portion of the population dependent on OOP payments or meager public services.	Studies of insurance schemes in Uganda and Tanzania highlight low enrollment and retention rates among informal workers due to income volatility.	(47, 48)

## 7 Policy implications

Policy approaches to strengthen health financing in LMICs are most effective when pursued as a deliberate sequence rather than a simultaneous wish-list, since early reforms create the conditions on which later reforms depend. Five priorities follow in order.

First, reducing point-of-care payment is the most urgent priority, because financial protection requires a decisive shift from OOP payments to compulsory prepayment and broad risk pooling. Globally, 1.4 to 1.9 billion people experience catastrophic or impoverishing health expenditures, largely in LMICs where regressive OOP payments dominate (2, 40). Systematic reviews show that public insurance generally increases utilization and often reduces catastrophic spending, although effects vary by coverage, benefit depth, and subsidization of poor and informal workers (2, 27). Evidence from Southeast Asia indicates that universalist approaches relying on general revenues, with mandatory inclusion and full subsidies for informal workers, achieve higher coverage and more sustained reductions in OOP expenditure than contributory, segmented schemes (8, 15). This shift depends on expanded public financing, since public funds already form the backbone of health financing but remain below the levels UHC requires; prioritizing health in budgets, improving tax administration, and adopting progressive instruments such as payroll contributions and earmarked levies are widely recommended to expand fiscal space (4, 15).

Second, defining an explicit and affordable benefit package links additional resources to clearly defined entitlements, supports dialogue with ministries of finance, and translates political commitments into medium-term budget appropriations (15, 41). A defined package also anchors decisions about what pooled funds will purchase.

Third, consolidating or harmonizing fragmented pools is necessary for sustainability and equity. Fragmented arrangements with separate schemes for formal workers, the poor, and community initiatives create multiple pools, varying benefits, and high administrative costs (2, 37). Reviews consistently show that fragmentation and voluntary enrollment are misaligned with UHC because they limit cross-subsidization. Consolidated systems funded from general revenues and mandatory contributions, with full subsidies for the poor and a unified benefit package, more reliably achieve equitable access than patchworks of voluntary schemes. Where consolidation is not feasible, harmonizing benefits, subsidies, and purchasing rules is a pragmatic interim step.

Fourth, strengthening strategic purchasing improves efficiency and equity within constrained budgets. Reforms have the greatest impact when accompanied by explicit benefit packages, provider accreditation, and payment methods such as capitation, diagnosis-related groups, and global budgets (15). Performance pay adds relatively little beyond simpler direct facility financing coupling operating budgets with provider autonomy and transparency [50]. Purchasing is most effective through unified national purchasers offering a common benefit package.

Fifth, reinforcing governance and public financial management is the foundation sustaining the rest. Governance reforms show the strongest evidence where they target financing, private partnerships, and community participation, and require clear regulation and credible contract management. Persistent OOP payments under free schemes often reflect provider coping with delayed reimbursement and resource shortages, so reforms must address frontline incentives, not only formal rules.

Together these priorities are mutually reinforcing rather than independent (Table 4), placing LMICs on a sustainable path toward UHC.

**Table 4:** Policy strategies to strengthen health financing for Universal Health Coverage.

Policy action	Required institutional conditions	Possible risks	Indicators for monitoring progress
Reduce point-of-care payment for essential services (tax-funded or subsidized)	Adequate public revenue; defined exempt services; provider reimbursement to replace lost fees	Facility underfunding if fees removed without replacing revenue; informal charges persist	OOP share of total health expenditure; incidence of catastrophic and impoverishing expenditure
Define an explicit, affordable benefit package	Costing and priority-setting capacity; dialogue with ministry of finance	Package too broad to fund, or skewed away from primary care	Share of population entitled to defined package; primary-care and high-burden service coverage
Consolidate or harmonize fragmented pools	Legal mandate; unified enrolment and information systems; political agreement across schemes	Resistance from existing schemes; stalled consolidation leaving parallel pools	Number of separate pools; share of population in the largest pool; cross-subsidization
Strengthen strategic purchasing	Provider accreditation; claims systems; purchaser capacity to set and monitor payment	Provider gaming, under-provision or over-treatment; cost escalation	Payment method mix; claims processing time; service utilization and quality measures
Strengthen governance and public financial management	Program-based budgeting; predictable disbursement; expenditure tracking; accountability mechanisms	Elite capture; weak enforcement; funds not reaching facilities	Budget execution rate; expenditure-tracking results; transparency and audit indicators
Integrate and align donor financing	Coordination mechanisms; co-financing rules; on-budget integration	Donor funds crowd out domestic allocation; fragmentation if off-budget	Donor share of health spending; proportion of external funds on-budget

## 8 Limitations

This review has several limitations that should be considered when interpreting its findings. As a narrative review, the study was not conducted under a registered protocol and did not employ a fully reproducible search string, a formal risk-of-bias assessment, or a systematic grading of evidence strength. The purposive approach to source selection, while appropriate to the narrative method, introduces a risk of selection bias, since the inclusion of sources depended partly on author judgement of relevance. The restriction to English-language publications may have excluded relevant evidence from non-English-speaking settings, and the review is also subject to possible publication bias, given that published studies tend to report more definitive findings. A further limitation is the review’s reliance on secondary syntheses such as systematic and narrative reviews, which may reduce the directness of the evidence and raises the possibility of double counting, where the same countries or programmes are represented both in primary studies and in the reviews that aggregate them. In addition, the considerable variation in health system contexts, institutional capacities, and socioeconomic conditions across LMICs means that findings may not be directly generalizable to all settings, and the depth of available evidence varied across the financing mechanisms examined, with innovative and earmarked financing strategies remaining comparatively understudied. These limitations notwithstanding, the manuscript provides a broad, policy-oriented synthesis of current evidence on health financing strategies for UHC in LMICs, whose conclusions should be interpreted in light of its narrative methods and the heterogeneity across LMIC settings.

## 9 Conclusion

This review’s central finding is that progress toward Universal Health Coverage in LMICs is unlikely under financing systems dominated by out-of-pocket payment, voluntary insurance, and fragmented risk pools, because these arrangements limit cross-subsidization and leave large population segments without effective protection. More plausible pathways involve compulsory public financing, subsidized coverage for poor and informal populations, larger and more unified risk pools, and purchasing arrangements that link payment to equity, quality, and efficiency. The evidence further suggests that the recurring difficulty of contributory social health insurance in settings with large informal sectors is structural rather than incidental, since payroll-based contributions cannot reach the majority where formal employment is limited, making tax-financed and subsidized coverage the more viable route.

These pathways should be understood as conditional rather than guaranteed. Financing reform is necessary but not sufficient for UHC, because service readiness, workforce capacity, medicine availability, and governance ultimately determine whether financial coverage becomes effective coverage. Recognizing these conditions, rather than prescribing a single universal model, offers the most credible foundation for equitable and sustainable financing reform across diverse LMIC settings.
